# Barriers and enablers to healthcare access for older adults in Cambodia: perspectives of healthcare professionals – a qualitative study

**DOI:** 10.1136/bmjopen-2025-101776

**Published:** 2025-09-30

**Authors:** Khin Thiri Maung, Socheata Phou, Monica Hunsberger, Ailiana Santosa, Nawi Ng, Heng Sopheab, Chhorvann Chhea, Malin Eriksson

**Affiliations:** 1 School of Public Health and Community Medicine, Institute of Medicine, Sahlgrenska Academy, University of Gothenburg, Gothenburg, Sweden; 2 National Institute of Public Health, Phnom Penh, Cambodia; 3 Department of Social Work, Umeå University, Umeå, Sweden

**Keywords:** Health Services Accessibility, PUBLIC HEALTH, Health policy

## Abstract

**Abstract:**

**Objective:**

To explore health professionals’ perspectives on the barriers and enablers of healthcare access for older adults in Cambodia.

**Design:**

A qualitative study based on semi-structured interviews conducted in Khmer, recorded, transcribed, translated into English and analysed using an abductive thematic analysis approach.

**Setting:**

Phnom Penh, Cambodia.

**Participants:**

A purposive sample of 11 health professionals serving in diverse roles and sectors participated in the study.

**Results:**

Three key barriers emerged: (1) institutional barriers, (2) patient-specific access barriers and (3) communication barriers. However, four key enablers were also identified: (1) supportive healthcare environment, (2) reaching out to improve access to health services, (3) peer and community engagement and (4) government direct support to access healthcare. Despite previous policy efforts, gaps in the implementation of healthcare services for older adults persist across all health facilities. Health professionals identified that improving healthcare access for older adults in Cambodia requires a multifaceted strategy involving proactive outreach, health promotion, financial assistance and stronger community and family support.

**Conclusion:**

Effective policy implementation requires collaboration among stakeholders and the active involvement of older adults in programme design to enhance dignity and well-being in Cambodia’s ageing population.

**STRENGTHS AND LIMITATIONS OF THIS STUDY**

- Purposive sampling captured health professionals from diverse roles and healthcare settings.
- Joint analysis by a native Cambodian researcher and an external researcher enhanced cultural and contextual interpretation.
- Rigorous translation and verification procedures minimised language-related inaccuracies.
- Daily research team debriefings and field notes supported data quality and consistency.
- Recruitment through National Institute of Public Health networks may have introduced selection bias and limited the transferability of the results.

## Introduction

 As the global population ages, understanding the complexities of accessing healthcare is increasingly important for maintaining physical function and overall well-being.^1^,^3^ Failure to address the factors that challenge access to healthcare can lead to unattainable, inadequate or delayed care, resulting in poorer health outcomes.^4^ Older adults represent a vulnerable demographic with more significant healthcare needs than other population groups, but they often face significant obstacles in accessing healthcare services globally.^5^ Therefore, understanding both barriers and enablers to accessing and providing essential care is a critical first step towards ensuring older people receive the timely and appropriate care they need.^6^

The WHO Study on global AGEing and adult health highlights the public health sector’s inability to adequately meet the needs of older adults, particularly in low- and middle-income countries (LMICs).^7 8^ In LMICs, older populations face significant barriers to healthcare access, which are compounded by economic, social and healthcare system challenges, including limited fiscal resources, inadequate infrastructure and a shortage of qualified healthcare professionals, as exemplified in research in Southeast Asia,^9^ Cambodia^10^ and Nigeria.^11 12^ Similar to many other LMICs, Cambodia contends with a dual burden of communicable and non-communicable diseases,^10^ making it challenging to manage prevalent conditions among its ageing population.^13^ Two studies conducted in Cambodia focused on the access to healthcare among older adults. The first study investigated access to oral healthcare^14^ and the second, to eye care,^15^ both identified financial constraints, transportation difficulties and lack of social or family support as critical factors affecting healthcare accessibility and acceptability.

Understanding the current state of healthcare access for older adults aged 50 and above, who comprise 13% of the Cambodian population^16^ is essential for policymakers and stakeholders to develop culturally relevant healthcare policies for older adults.^9^ Identifying barriers to access can help tailor strategies that improve healthcare delivery.^17^,^19^ There is a scarcity of studies focused on healthcare access for older adults in Cambodia, particularly from the perspective of health professionals. This study contributes to the existing knowledge gap by exploring healthcare professionals’ perspectives on the barriers and enablers to healthcare access in Cambodia.

## Methods

### Study population and design

This qualitative interview study was conducted in Phnom Penh, Cambodia, with 11 health professionals. We used a qualitative study design to capture participants’ experiences and viewpoints, employing a semi-structured interview guide to allow them to express themselves freely and provide in-depth perspectives. Participants were recruited using a purposive sampling method which aimed to capture a heterogenous group of health professionals, including nurses, doctors, midwives, pharmacists, traditional medicine practitioners and representatives from national government institutions and non-government organisations (NGOs). Potential participants were identified through networks facilitated by the National Institute of Public Health (NIPH), Cambodia and were invited to participate through targeted outreach efforts. With purposive selection, the aim was to enlist both men and women in diverse roles across various healthcare settings to capture a broad understanding of healthcare professionals’ perceptions regarding the challenges faced by older adults while ensuring the findings reflected a wide range of experiences and factors influencing their access to and utilisation of healthcare services. Interviews were conducted at participants’ preferred locations, primarily their workplaces, including district referral hospitals, private clinics and health centres.

### Data collection

All participants were provided with information about the study and then signed an informed consent form before the interview. Interviews were conducted between September and October 2023 using a semi-structured interview guide which was developed based on previous literature and then pilot-tested with three health professionals (a pharmacist, a midwife and a nurse). After pilot testing, no revisions were deemed necessary, and the pilot interview data were included in the final analysis. The interviews were conducted in Khmer by an experienced interviewer with prior involvement in NIPH research projects. The interviewer was accompanied by a notetaker, specifically recruited and trained for this qualitative study.

In-person interviews were conducted with nine participants, while the remaining two participants were interviewed by phone, as per their preferences. The research team held daily meetings throughout the data collection period to maintain project integrity, review findings and identify areas for further probing in the subsequent interviews. All interviews were audio-recorded, with an average duration of approximately 60 min. In addition to the recordings, field notes were taken during the interviews to document factors that could affect the flow and quality of the interview session and to identify areas that might require further elaboration.

### Data analysis

The recorded interviews were transcribed verbatim in Khmer, anonymised to ensure confidentiality and then translated into English. The translation was carried out by a team comprising one researcher, the second author (SP) and six external translators proficient in both Khmer and English. Once the translation was completed, SP reviewed the translated text alongside the original Khmer text to ensure accuracy and consistency. Any inconsistencies identified were resolved through discussion with the translation team until consensus was achieved. Additionally, the first author (KTM) reviewed the English translations and collaborated with SP to address any ambiguities. If any issues arose, both authors cross-checked with the Khmer version for clarity.

We employed thematic analysis with an abductive approach, inspired by Braun and Clarke.^20^ A predetermined thematic framework, derived from the study’s objectives, guided the analysis, while we also considered the distinctive contextual perceptions of Cambodia’s healthcare professionals and remained open to unanticipated subthemes. Microsoft Word and Excel were used for coding the transcripts and managing the data. Braun and Clarke^20^ propose six distinct phases in the analysis, while also acknowledging the flexibility in analysing qualitative data. In the first step, and in line with Braun and Clarke,^20^ KTM and SP independently familiarised themselves with the data, identified keywords and developed initial codes. Then, both authors (KTM and SP) engaged in discussions and compiled the agreed-upon codes along with their defining statements. The codes were then sorted and used to identify subthemes and overarching themes at a semantic and descriptive level. Illustrative quotes were selected to support each theme.

The analysis process benefits from the diverse perspectives of the authors (KTM and SP): KTM brought an outsider viewpoint, while SP, a native Cambodian, provided cultural and contextual insights. This collaborative approach facilitated a richer and more nuanced interpretation of the data. The remaining research team subsequently reviewed the thematic analysis and provided feedback to refine the interpretation of the findings.

### Patient and public involvement

Patients and the public were not involved in the design, conduct, reporting or dissemination plans of this research.

## Results

### Study participants

As shown in table 1, six of the 11 participants were women. The mean age was 44 years (ranging from 28 to 59 years). Work experience ranged from 2.5 to 36 years, with seven participants having over 15 years of work experience. The interviewees consisted of six health professionals from the public sector, four from the private sector and one from a NGO.

**Table 1:** Characteristics of health professionals (n=11)

Characteristics	n (%)
Gender	
Female	6 (55.0)
Male	5 (45.0)
Working experience	
>15 years	7 (63.6)
<15 years	4 (34.4)
Sectors	
Private	4 (36.3)
NGO	1 (9.1)
Public	6 (54.5)
Age in years (min–max)	28–59

NGO, non-government organisation.

Participants’ perspectives on healthcare access for older adults in Cambodia were grouped into two main themes: (1) barriers to accessing healthcare and (2) enablers to accessing healthcare (table 2). While perspectives on barriers and enablers were largely consistent across the public and private sectors, some minor differences in attitudes among healthcare providers were observed. Quotations were attributed to participants’ workplaces and study IDs to provide context.

**Table 2:** Codes, subthemes and themes from the thematic analysis

Examples of codes	Subthemes	Themes
Knowledge gap in health policy translation	Institutional barriers	Barriers to accessing healthcare
Limited capacity of healthcare providers		
Lack of outreach services for older people		
Lack of screening service, equipment and medicine		
Lack of money to pay for healthcare-related services	Patients’ specific access barriers	
Difficult to transport to healthcare services		
Lack of family support to get healthcare		
Insufficient knowledge of where to access healthcare		
Disrespectful behaviours of healthcare providers to older people	Communication barriers	
Lack of positive interaction between the provider and the clients		
Financial support from providers, such as payment options for healthcare services	Supportive healthcare environment	Enablers to accessing healthcare
Friendly environment		
Easing accessibility to healthcare services	Reaching out to improve access to health services	
Raising awareness about the services offered		
Involve community groups in promoting healthcare services	Peer and community engagement	
Peer pressure to get healthcare services		
Eligibility for a financial need identification card	Government direct support to access healthcare	

### Barriers to healthcare access by older adults

Three subthemes emerged under the main theme barriers to accessing healthcare as follows: (1) institutional barriers, (2) patient-specific access barriers and (3) communication barriers. Together, these subthemes captured the range of challenges older adults faced when seeking healthcare and the specific constraints that limit their access to needed services.

#### Institutional barriers

At the health facility level, the lack of policy implementation, limited capacity of healthcare providers, insufficient outreach activities and the absence of screening services for communicable and non-communicable diseases, as well as equipment and essential medicines were identified as barriers to older adults accessing healthcare. Most health professionals were unaware of healthcare policies for older adults. Among the participants who were aware of these policies, they claimed that these policies had not been integrated into routine primary care practices at their facilities. They emphasised that the absence of mandates for policy implementation hindered the provision of healthcare services for older adults across all health facilities.

*At the national level, healthcare policies have been developed, but there is a lack of information at the sub-national level. Additionally, while policies exist, in some cases there is no action plan, monitoring or evaluation system.* (IDI007 - public sector)

Health professionals disclosed their inability to provide adequate healthcare services for older adults within their facilities due to a shortage of skilled healthcare providers. A consistent theme throughout the interviews was the significant challenge posed by the limited number of qualified health providers at health centres.

*The staff has limited capacity and technical skills in providing healthcare services for older adults. While there may be an adequate number of staff, the absence of medical doctors is notable.* (IDI002 - public sector)

Some health professionals acknowledged the challenges older adults face, particularly mobility limitations and disability, which were exacerbated by the lack of government-supported outreach services. The absence of clear plans or guidelines for outreach efforts posed significant barriers, hindering improvement in community healthcare access and outcomes.

*Older adults experience disability problems, and the government lacks outreach services. Without outreach activities from health centers to promote community health, access to healthcare services will remain low.* (IDI010 - NGOs)

Health professionals expressed frustration over the inadequate screening services and their inability to meet the needs of older patients effectively. This was attributed to a lack of laboratory facilities and persistent shortages of essential medications.

*The national hospital advises them (older people) to take medicine at their nearest health center, but our health center doesn’t have the medicine they need. We want to provide what they need, but we don’t have it available.* (IDI003 - public sector)

#### Patients’ specific access barriers

Most health professionals observed that financial constraints, living in remote areas, limited awareness of available services and reliance on family decision-making hindered older adults’ access to healthcare.

All health professionals highlighted the significant impact of financial constraints on older patients. These constraints included the inability to afford medical expenses and the challenges in covering transportation costs and other related expenditures. Consequently, older adults may prioritise other needs over accessing healthcare, leading to delayed or forgone medical treatment.

*The service fee is not the problem; it is the traveling costs. Some patients travel from Koh Kong to Phnom Penh for their appointments. They spent around a day traveling to Phnom Penh, and sometimes they stay overnight at their relatives’ homes. They incur costs for a round trip. The service fee at the center is only 40 000 riels [around $10], which is not a problem. The main financial challenges are travel and medicine costs.* (IDI007 - public sector)

Geographical challenges further compounded access, particularly for older adults residing in remote or rural areas. Health professionals frequently witnessed firsthand the obstacles patients encountered in reaching healthcare facilities far from their homes. Long travel distances and limited transportation options exacerbated difficulties seeking timely medical care.

These barriers were especially pronounced for older adults who lived alone, those who lacked family support and those with limited mobility or a health-related disability.

*Even if a healthcare facility is nearby, it doesn’t necessarily mean it’s easy to access. Some are disabled and they struggle to travel to another location.* (IDI006 - public sector)

Health professionals also noted that older patients often relied heavily on family members to make decisions about their healthcare. This reliance sometimes led older adults to forgo necessary care when family members deemed it unnecessary.

*Some (family members) thought it was not necessary to bring them (older people) to the hospitals and chose to take care of them at home until they passed away.* (IDI007 - private sector)

Most health professionals described the critical role of health literacy in shaping healthcare-seeking behaviour among older patients. A lack of knowledge about medical conditions, available healthcare services and treatment options hindered older adults from making informed decisions about their health and access to healthcare.

*They don’t know where they can access healthcare services or health information.* (IDI010 - private sector)

#### Communication barrier between healthcare providers and older people

Some health professionals elaborated on feelings of fatigue and stress, expressing frustration over managing a high patient volume. This heavy workload adversely affected their ability to communicate effectively with patients, leading to situations where patients perceived providers as uncaring or rude due to unanswered questions or limited engagement.

The health professionals noted that insufficient positive interaction and unclear explanations from doctors to patients were more prevalent in public hospitals. This led to misunderstandings and dissatisfaction among patients, deterring them from seeking healthcare in the future.

*When they (the older people) do not understand what the doctors explain, they no longer come to the hospitals.* (IDI008 - private sector)

Public sector health professionals admitted that they might unintentionally exhibit such behaviour due to stress. Many older adults felt scared or nervous when communicating with healthcare providers, which sometimes led to withholding information or avoiding care altogether.

*It’s common that when we’re stressed, we may become unfriendly with them. Some patients also caused trouble for us. Sometimes, they were troublesome. I’ve experienced it too [Laugh].* (IDI002 - public sector)

NGO professionals noted that poor interactions with healthcare providers discouraged older adults from seeking necessary medical services.

*They (the older people) hesitate to seek health services at health centers because they fear being blamed by healthcare workers.* (IDI010 - NGOs)

### Enablers to healthcare access for older adults

Four subthemes emerged under the main theme of enabling access to healthcare for older adults. These included (1) creating a supportive environment to access healthcare, (2) reaching out to improve access to health services, (3) peer and community engagement and (4) government support. These subthemes collectively addressed health professionals’ views on the various enablers for older adults to access healthcare services, offering comprehensive strategies to improve their overall healthcare access and outcomes.

#### Supportive healthcare environment

Many health professionals highlighted that a supportive environment comprised various elements that collectively enhance adults’ ability to access and use healthcare services. They shared their experiences, emphasising that when patients feel familiar and comfortable with their healthcare providers, they are more inclined to seek healthcare services without hesitation, even in challenging circumstances. This sense of familiarity fostered a welcoming and friendly environment, further encouraging the use of healthcare services. Moreover, a supportive environment addressed practical barriers, such as financial constraints, by offering flexible payment options and financial assistance tailored to the needs of financially constrained older adults. By minimising financial obstacles, health professionals ensured a supportive environment in which medical care is accessible, enabling adults to seek necessary healthcare services without economic hindrance.

Health professionals described how they engaged older people in accessing healthcare by offering flexible payment options when they are unable to afford the service fees. They also noted that this kind of financial support is only available at the public health facilities.

*We don’t ask them (the older people) to pay. If they are poor and can’t pay, we write in the record as an exceptional case.* (IDI003 - public sector)

Most health professionals observed that patients were more likely to seek and access healthcare services once they became familiar with their healthcare providers.

*They feel familiar with the healthcare providers who came to their community for health campaigns or outreach activities. This familiarity also encourages them to go to the health center where these providers have been assigned.* (IDI010 - NGOs)

#### Reaching out to improve access to health services

Health professionals stressed the importance of proactive outreach and ensuring healthcare services were easily accessible to all. This involved actively engaging with communities to raise awareness and educate people about available healthcare resources. Making healthcare services accessible included establishing healthcare facilities and providing transportation options to reduce the barriers older people faced in accessing healthcare. Additionally, it was essential to educate caregivers and older adults about the importance of seeking timely healthcare when needed.

Some health professionals highlighted that when hospitals, clinics, pharmacies and health centres were in areas where older people frequently visited and could easily reach, it enhanced their healthcare utilisation.

*Most of the older adult patients who came to this health center live nearby.* (IDI005 - public sector)

Health professionals expressed their efforts to raise awareness among older people and their caregivers regarding available healthcare resources and how to access the healthcare system more effectively. They emphasised the importance of seeking proper medical treatment, especially for severe illnesses, to ensure that older adults received timely and appropriate care for their healthcare needs.

*We provide training to the older adults to enable them to access essential services.* (IDI010 - NGOs)

#### Peer and community engagement

Health professionals emphasised the crucial important role of peer and community engagement in enhancing healthcare access for older adults. These initiatives helped raise awareness of available healthcare services, empowering older adults to navigate the healthcare system with confidence.

Health professionals emphasised the influence of peers sharing their experiences and recommendations about healthcare providers and treatment options. When prospective patients witness positive outcomes, they become natural advocates for the healthcare facilities, encouraging others to seek care. Peer endorsement fostered trust and a sense of community, leading to increased healthcare utilisation.

*When they first came, some people did not trust us, but once some tried, and they started to feel better, others noticed, and they too came to give it a try.* (IDI009 - private sector)

The health professionals from the private sector noted their collaboration with the Ministry of Health in establishing community groups, a successful initiative implemented across eight provinces. These groups are tasked with addressing healthcare challenges within their communities and advocating for the promotion of healthcare services.

*The village volunteers organized a health campaign with us in the community. They have been trained on how to explain to the poor people in the village the importance of seeking and utilizing healthcare services.* (IDI003 - public sector)

#### Government direct support to access healthcare

Most health professionals explained that patients with a Poor ID or National Social Security Fund (NSSF) card could access services either free of charge or pay a small amount of user fee. Participants noted that patients with low socioeconomic status often used these cards when seeking care.

*Patients with low socioeconomic status from the provinces use Poor ID when accessing services at our center after initially using primary health facilities.* (IDI007 - public sector)

However, participants also mentioned that this type of public insurance can only be used in public hospitals and health centres. The private sector does not accept this card.

*The public hospitals will certainly accept to use of this ID card, but the private hospitals most rarely accept it.* (IDI008 - private sector)

## Discussions

In this study, we explored the perspectives of health professionals on barriers and enablers to healthcare access for older adults in Cambodia. In this qualitative study, we interviewed informants from the public and private sectors, as well as from NGOs. The Cambodian National Health System reviews indicate that the public sector plays the predominant role in planning and delivering health services.^21^ In contrast, the private sector and NGOs contribute to a lesser extent, yet still significantly. Despite their role in supporting the implementation of the government’s policies, the perspectives of private providers and NGOs are often under-represented in the policymaking process. Our study, therefore, offers valuable new insights to challenges faced by the public sectors in providing healthcare for older adults in Cambodia, and how the private sectors and NGOs could support the implementation of the service provision. The inability of local healthcare providers to deliver necessary treatment and screening services forces older adults to travel to distant national or provincial hospitals, exacerbating their financial burden. This finding aligns with previous studies conducted in Cambodia^13^ and Bangladesh,^22^ and similar challenges have been noted even in Malaysia’s highly subsidised healthcare system.^23^ Prioritising initiatives, such as establishing well-equipped health centres with trained professionals in elderly care^24 25^ and implementing outreach activities,^26^ is crucial to addressing these challenges related to the availability and accessibility of care needed by the older population.

Despite having well-outlined strategies aimed at addressing healthcare access challenges, these policies have not been fully implemented, perpetuating the challenges faced by older adults. We found a significant gap in health professionals’ awareness of existing policies and strategies for older adults such as the National Policy on Ageing (2017–2030)^27^ and the National Healthcare Policy and Strategy for Older People (2016),^28^ leading to uncertainty about their roles and responsibilities in delivering healthcare services. Similar concerns were reported in a study from Ethiopia, highlighting the need for strategic planning to ensure healthcare providers have the necessary resources.^29^ WHO highlights a related issue, emphasising that adequate human resources for health initiatives necessitate the collection, processing and dissemination of relevant data, as well as its utilisation in policy and managerial decisions. It advocates for the establishment of health workforce observatories, which help bridge the gap between evidence and policy by offering cooperative mechanisms for countries to share information and improve healthcare practices.^30^ This highlights the need for improved communication and resource allocation, which could address the gaps in awareness and implementation identified in our study.

It is well known that these barriers primarily affect older adults who are economically disadvantaged, have low health literacy and lack family support,^31^ which aligns with the findings of this study. Moreover, disrespectful attitudes and inaccessible interaction with healthcare providers emerged as significant barriers to healthcare utilisation among older adults in this study. Health professionals in the public sector often attributed these issues to service overload. In contrast, those from the private and NGO sectors acknowledged that older adults frequently felt that providers were unresponsive, disrespectful and insensitive. Although this study did not investigate the reasons behind these differing perspectives, previous study has shown that older people often value compassionate care more than extensive medical interventions from healthcare professionals.^32^ When they do not receive the expected care and respect, it leads to dissatisfaction and misunderstandings regarding their medical care, potentially discouraging them from seeking future healthcare. This aligns with previous findings from both high-income and LMICs. For example, in the USA^33^ and Namibia,^34^ the most commonly reported barriers were healthcare providers’ lack of responsiveness and poor attitudes. These findings suggest that improving healthcare providers’ attitudes could enhance formal healthcare utilisation among older adults, particularly those who are economically disadvantaged.

As part of government support, the Health Equity Fund (HEF) serves as a demand-side financing mechanism providing user fee exemptions for identified low-income individuals accessing government health facilities, and directly reimbursing these facilities for the services rendered.^35^ Government-issued identification cards to low-income households and established a social registry for poor and vulnerable families, enabling targeted access to healthcare.^36^ The scheme covers user fees for all services at various levels of public healthcare facilities, encompassing a basic treatment, a preventive care package at the health centre level and a complementary package at the referral hospital level to address more complex health issues, including surgical care. Additionally, poor patients receive non-medical benefits such as reimbursement of transportation costs to and from the referral hospitals, food allowances for caregivers and funeral support.^35^ The NSSF extends social security coverage to workers in both the formal and informal sectors, as well as self-employed individuals who have registered with the NSSF. This coverage includes medical expenses and income replacement benefits for illness or maternity-related absences from work,^37^ helping ensure that individuals who can afford healthcare costs still have access to necessary healthcare services. Currently, NSSF coverage has reached 44.5% of the population, with the intention of expanding to 80% by 2035.^38^ These support systems ensure that older people in need have increased opportunities to seek care at public health facilities.^39^ Consequently, individuals who possess these cards are more empowered to overcome financial barriers to access the healthcare services they require, ultimately leading to improved overall health outcomes. Despite these benefits, previous studies have shown that 36% of individuals living below the national poverty line remained uncovered by the HEF.^40 41^ The government must expand coverage and ensure that all older people, particularly the poorest, have access to these essential financial support mechanisms. Targeted efforts must be made to identify and include poor older adults who are currently excluded from these funds, in an attempt to promote equitable healthcare access for all.

Previous studies have highlighted the significant role of family support^19 42^ and the effectiveness of integrating community engagement services^43^ in facilitating older adults’ access to healthcare services. This study reinforces these findings, demonstrating that family involvement and community support are crucial for achieving better healthcare outcomes. Community leaders, such as village chiefs, play a crucial role in promoting healthcare services, while enhancing family support through caregiver education remains vital. The results highlight the importance of increasing caregiver awareness and motivation to adhere to recommended healthcare strategies within supportive community networks.

Moving forward, effective policy implementation and meaningful engagement are vital and require the collaboration among policymakers, healthcare providers, community organisations and other stakeholders. Active involvement of older adults in programme development is also essential. A multifaceted approach is necessary to enhance healthcare accessibility, including proactive outreach, promoting of healthcare services through various media and establishing financial support programmes. Integrating these approaches can effectively address barriers to healthcare access and improve health outcomes for older populations.

### Strengths and limitations

This study’s strengths lie in its purposive sampling approach, which focused on health professionals in diverse settings, including public and private healthcare sectors, government institutions and NGOs. This approach enabled in-depth insights from frontline service providers and individuals actively involved in the care of older adults. As a result, the findings offer valuable perspectives that can inform the development and improvement of healthcare services for older adults and programmes tailored to their specific needs. However, the study’s focus on health professionals in the capital city limits the diversity of perspectives, as it may not capture the full range of views from health professionals across different regions.

## Conclusion

Health professionals in Cambodia perceived that the Cambodian healthcare system continues to face significant challenges in meeting the needs of older adults. Although our study did not identify unexpected barriers or enablers within the Cambodian context, it did highlight the insufficient implementation of existing policies, such as the National Health Care Policy and Strategy for Older People (2016).^28^ By addressing the challenges identified in this study and implementing comprehensive, community-driven interventions, Cambodia can improve healthcare access and outcomes for older adults, ultimately promoting their well-being and dignity in later life.

### Recommendation for future research

While this study provides valuable insights from health professionals, several areas warrant further investigation to better support the ageing population in Cambodia and other LMICs. First, longitudinal research is needed to examine how barriers to healthcare access evolve among older adults. Second, comparative studies across different LMICs could help identify context-specific determinants of access and inform targeted interventions. Third, a mixed method approach that integrates quantitative analyses of healthcare utilisation with qualitative perspectives from patients and providers could yield a more comprehensive understanding of barriers and facilitators. In addition, future research should also explore the potential of community-based services in addressing existing gaps, examine the quality of life among older adults, investigate access to mental healthcare in ageing populations and assess the role of digital health innovations in overcoming infrastructure and workforce limitations. Finally, greater attention is needed to how social determinants such as gender, socioeconomic status and rural–urban residence shape healthcare access among older adults, to inform more equitable health policies and service delivery strategies.

## Data Availability

No data are available.
