# Supplementary material for: Barriers and enablers to healthcare access for older adults in Cambodia: perspectives of healthcare professionals – a qualitative study
Source: BMJ Open. 2025 Sep 30;15(9):e101776. doi: 10.1136/bmjopen-2025-101776 (PMC12496091; doi:10.1136/bmjopen-2025-101776)
Supplement: Supplementary file 1 [file bmjopen-2025-101776.reviewer_comments.pdf]

PEER REVIEW HISTORY

BMJ Open publishes all reviews undertaken for accepted manuscripts. Reviewers are asked to complete a checklist review form and are provided with free text boxes to elaborate on their assessment. These free text comments are reproduced below.

ARTICLE DETAILS

Title (Provisional)

A qualitative study of barriers and enablers to healthcare access for older adults in Cambodia: Perspectives of healthcare professionals

Authors

Maung, Khin Thiri; Phou, Socheata; Hunsberger, Monica; Santosa, Ailiana; Ng, Nawi; Sopheab, Heng; Chhea, Chhorvann; Eriksson, Malin

---

VERSION 1 - REVIEW

**Reviewer**	**1**
**Name**	**Pannarunothai, Supasit**
**Affiliation**	**Centre for Health Equity Monitoring Foundation**
**Date**	**12-Jul-2025**
**COI**	**None**

As the authors wrote in the conclusion that the paper did not identify unexpected barriers and enablers, the authors gave high value to the findings of insufficient implementation of existing policies. The study recruited 6 key informants from public sector, 4 from private sector and one from an NGO. The authors may consider adding discussions related to the proportions of key informants as according to proportions of key providers of services to older persons in Cambodia, and in relation to the gaps of policy implementations from public, private and NGO sectors.

Minor errors were found:

Referencing of Braun and Clarke should be 18 not 17.

Check spelling/typo

On page 15 of 25, line 46

On page 17 of 25, line 29

**Reviewer**	**2**
**Name**	**Omona, Kizito**

Affiliation **Uganda Martyrs University, Faculty of Health Sciences**
Date **19-Jul-2025**
COI **None**

Introduction: State the objective of the study at the end of the introduction section

Study Design: Precisely state the qualitative design used and the rationale for the choice of the design

Results: It would be better to remove all discussions from the results section and take them to the discussion section. Deal with results only in the result section.

Reviewer **3**
Name **Ogueji , Ifeanyichukwu Anthony**
Affiliation **University of Ibadan, Psychology**
Date **21-Jul-2025**
COI **None**

Thank you for the opportunity to review this importance piece of work. A thorough investigation of access barriers to health healthcare services was reported here and the findings are meaningfully presented. Nevertheless, I recommend some revisions to the authors:

- The second paragraph where this statement is made should also be supported with the following studies "In LMICs, older population face significant barriers to healthcare access, compounded by economic, social, and healthcare system challenges such as limited fiscal resources, inadequate infrastructure, and a shortage of qualified healthcare professionals (9,10).":

Ogueji, I. A., Ogunsola, O. O., Abdalla, N. M., & Helmy, M. (2024). Mistrust of the Nigerian health system and its practical implications: qualitative insights from professionals and non-professionals in the Nigerian health system. Journal of Public Health, 32(2), 303-314. https://doi.org/10.1007/s10389-022-01814-z

Ogueji, I. A., Ogunsola, O. O., Abdalla, N. M., & Helmy, M. (2024). Exploring health workers' perspectives on referral system challenges in Nigeria. Journal of Health Management, 09720634241263391. https://doi.org/10.1177/09720634241263391

These studies were conducted in Nigeria, a LMIC, and they report a comprehensive picture of the health system deficiencies, making them suitable to support your argument.

- The manuscript lacks directions for future research.

- In my own assessment, the authors have not convincingly communicated the novelty of their study. I encourage them to elaborate on the unique contributions that their study makes to knowledge.

Overall, this is an important work and I believe that if the reviewers' comments are addressed, the manuscript's contents will be more understandable.

Overall,

VERSION 1 - AUTHOR RESPONSE

Reponses to Reviewer 1

Comment (1):

The authors may consider adding discussions related to the proportions of key informants as according to proportions of key providers of services to older persons in Cambodia, and in relation to the gaps of policy implementations from public, private and NGO sectors.

Response (1):

We thank the reviewer for the suggestion. We respectfully disagree to add a discussion comparing the distribution of key service providers in Cambodia and our study. We, however, have taken the reviewer's perspective and have edited our discussion reflect the role of public and the private sectors as well as NGO in service provision in Cambodia. In qualitative research, representation typically involves purposive sampling to recruit participants with specific knowledge or experience of the phenomenon under study. Our study participants are resource person deemed to be knowledgeable on the topic of this study, and it has no relevance with the distribution of key providers of services for older people in Cambodia. Cresswell and Plano Clark discussed the issue of sampling and representativeness of qualitative study in their book. (Cresswell JW, Plano Clark VL. Designing and conducting mixed method research. 2nd Sage; Thousand Oaks, CA: 2011).

We have revised the Discussion section as follows (see *Discussion_Clean Version* manuscript, page 15, lines 315–324).

Comment (2):

Referencing of Braun and Clarke should be 18 not 17.

Response (2):

We have corrected the in-text citation for Braun and Clarke to reference number 20, after adding additional citations, and verified the numbers of all references in the manuscript.

Comment (3):

Check spelling/typo on page 15 of 25, line 46, and on page 17 of 25, line 29.

Response (3):

We have corrected these typo errors and reviewed the entire manuscript for any additional spelling mistakes.

Responses to Reviewer 2

Comment 1:

Introduction: State the objective of the study at the end of the introduction section

Response 1:

We sincerely thank the reviewer for this valuable comment. We have revised and stated the objective of the study at the end of the introduction section.

Comment 2:

Study Design: Precisely state the qualitative design used and the rationale for the choice of the design

Response 2:

We appreciate the reviewer's insightful suggestion. In the Methods section, we have specified the exact qualitative design employed and provided a clear rationale for choosing this design to strengthen the clarity of the study. Please refer to *Method _Clean Version* manuscript, page 5, lines 36-38.

Comment 3:

Results: It would be better to remove all discussions from the results section and take them to the discussion section. Deal with results only in the result section.

Response 3:

Thank you for your suggestion. We have moved most of the discussion previous included in the Result section, along with the corresponding references to the Discussion section. However, we have kept some texts in the Result section where it was necessary for clarity and interpretation of the findings.

Responses to Reviewer 3

Comment 1:

The second paragraph where this statement is made should also be supported with the following studies "In LMICs, older population face significant barriers to healthcare access, compounded by economic, social, a communication gap between health facilities and healthcare system challenges such as limited fiscal resources, inadequate infrastructure, and a shortage of qualified healthcare professionals (9,10).":

Ogueji, I. A., Ogunsola, O. O., Abdalla, N. M., & Helmy, M. (2024). Mistrust of the Nigerian health system and its practical implications: qualitative insights from professionals and non-professionals in the Nigerian health system. Journal of Public Health, 32(2), 303-314. https://doi.org/10.1007/s10389-022-01814-z

Ogueji, I. A., Ogunsola, O. O., Abdalla, N. M., & Helmy, M. (2024). Exploring health workers' perspectives on referral system challenges in Nigeria. Journal of Health Management, 09720634241263391. https://doi.org/10.1177/09720634241263391

These studies were conducted in Nigeria, a LMIC, and they report a comprehensive picture of the health system deficiencies, making them suitable to support your argument.

Response 1:

We thank the reviewer for recommending the papers which provide a comprehensive perspective on health system deficiencies and further support our argument. We have incorporated the suggested references into the second paragraph to strengthen our discussion of healthcare access barriers in LMICs.

Comment 2:

The manuscript lacks directions for future research.

Response 2:

We appreciate the reviewer's recommendation. We have now included "Directions for future research" as a section at the end of the Discussion section. In this section, we emphasize the need for further studies exploring intervention strategies to mitigate access barriers, as well as cross-country comparative research to deepen understanding of contextual differences.

Comment 3:

In my own assessment, the authors have not convincingly communicated the novelty of their study. I encourage them to elaborate on the unique contributions that their study makes to knowledge.

Response 3:

We are grateful for this insightful comment. We have expanded our explanation of the novelty of the study which clarifies the distinct contribution our study makes to knowledge.

After revising the manuscripts according to the responses from editor(s) and reviewers, we believe that our manuscript has significantly strengthened than the previous version. We are grateful to the editor(s) and reviewers for their constructive input, which has improved the clarity, rigor, and relevance of our work. We respectfully resubmit the revised manuscript for your kind consideration.
